# A woman with cystic fibrosis, severe hypoxaemia, an atrial thrombus and a patent foramen ovale: a case report

**DOI:** 10.4076/1752-1947-3-8582

**Published:** 2009-07-20

**Authors:** Nicholas J Simmonds, Hilary Wyatt, Raj Patel, Margaret E Hodson, Khin M Gyi

**Affiliations:** 1 Department of Cystic Fibrosis, Royal Brompton Hospital and NHLI/Imperial College, Sydney Street, London, SW3 6NP, UK; 2 Department of Cystic Fibrosis, King's College Hospital, Denmark Hill, London, SE5 9RS, UK; 3 Department of Haematology, King's College Hospital, Denmark Hill, London, SE5 9RS, UK

## Abstract

**Introduction:**

Cystic fibrosis is usually associated with chronic pulmonary sepsis and frequent infective exacerbations. We report a very unusual cause of severe hypoxaemia in a woman with cystic fibrosis caused by thrombus formation in the right atrium.

**Case presentation:**

A 21-year-old Caucasian woman with cystic fibrosis and a totally implantable venous access device presented with severe hypoxaemia. This was initially treated with antibiotics but her oxygen levels did not improve significantly. Subsequently, a transient ischaemic attack occurred. Further investigations, including a contrast echocardiogram and a cardiac magnetic resonance scan, revealed the presence of a large right atrial thrombus and right-to-left intracardiac shunt through a patent foramen ovale.

**Conclusion:**

This case highlights the need to consider a right-to-left shunt in chronic respiratory diseases when hypoxaemia is out of proportion to the degree of lung function impairment. Totally implantable venous access devices should always be considered as a source of thrombus formation.

## Introduction

Cystic fibrosis (CF) is an autosomal, recessively inherited disease caused by mutations in the cystic fibrosis transmembrane conductance regulator (*CFTR*) gene. It is common in Caucasian populations, giving rise to an incidence of approximately 1 in 2000 newborns. It has a predilection for the lungs and gastrointestinal tract, commonly manifesting as bronchiectasis and malabsorption secondary to pancreatic insufficiency. Progressive hypoxaemia occurs as a result of pulmonary sepsis and associated obstruction of the airways [1]. Despite being a chronic inflammatory condition with a purported increased incidence of thrombophilia [2,3], thromboembolic disease is rarely reported except in association with totally implantable venous access devices (TIVADs) [4]-[8] which are often inserted when frequent courses of intravenous antibiotics are required.

We describe a patient where a number of seemingly unrelated symptoms shared a mutual aetiology, presenting with severe hypoxaemia that was disproportionately low for her magnitude of pulmonary disease; investigations revealed a right-to-left intracardiac shunt as a result of a patent foramen ovale (PFO) and a right atrial thrombus.

## Case presentation

A 21-year-old Caucasian woman with CF, rheumatoid arthritis (RA) and a TIVAD presented with deterioration in her oxygen saturation, from a baseline of 95%, to 88% in room air. This was attributed to an infective exacerbation as her forced expiratory volume in 1s (FEV_1_) had decreased from 70 to 50% predicted. Arterial blood gas assessment revealed a pO_2_ of 5.8 kPa and a pCO_2_ of 4.1 kPa in room air. The diffusing capacity of carbon monoxide corrected for loss of alveolar volume (KCO) was 60% predicted. A thoracic computed tomography (CT) scan (including high resolution cuts and pulmonary angiography) revealed moderate bronchiectasis, small airways disease and mucus plugging with no evidence of pulmonary embolism. Her routine medications included nebulised preservative-free tobramycin (TOBI; Novartis, Basel, Switzerland), oral flucloxacillin, azithromycin, prednisolone (variable dose), multivitamins, vitamin E, calcium tablets, zoledronic acid, budesonide/formoterol inhaler, terbutaline inhaler, pancreatic enzymes, insulin glargine/lispro and naproxen. She was relatively immobile due to her RA and so did not complain of significant breathlessness. Intensive treatment with intravenous antibiotics and physiotherapy improved her FEV_1_ to baseline but her oxygen saturation only partially corrected (90% in room air).

**Figure 1:**
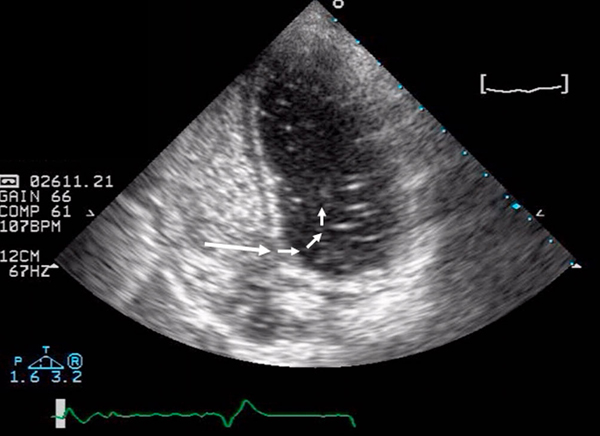
**Contrast ('bubble') transthoracic echocardiogram showing the presence of a patent foramen ovale (large arrow) with a right-to-left shunt (small arrows)**.

**Figure 2:**
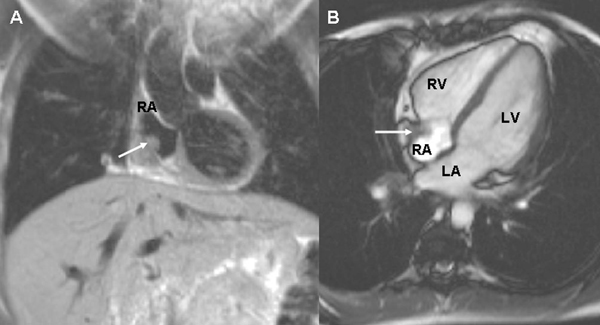
**Cardiac magnetic resonance imaging showing the position of the thrombus (arrow) in coronal view (A)** and transverse view **(B)**. RV = right ventricle, LV = left ventricle, LA = left atrium and RA = right atrium. Courtesy of Dr Raad Mohiaddin.

One month later, she presented with transient expressive dysphasia and a right-sided hemiparesis. Oxygen saturation was 85% in room air and blood tests revealed the presence of lupus anticoagulants but anticardiolipin antibodies were negative (Table 1). Brain CT, lumbar puncture and carotid dopplers were all normal. The neurology resolved within 24 hours and a transient ischaemic attack (TIA) was diagnosed. A transthoracic echocardiogram revealed a right atrial mass (there was no evidence of intracardiac shunting or abnormal right heart pressures). Further evaluation with a contrast echocardiogram (Figure 1) and cardiac magnetic resonance imaging (Figure 2), confirmed the presence of a right atrial thrombus close to the distal end of her TIVAD and also a PFO with right-to-left shunting. A physiological shunt study was also performed, confirming a shunt fraction of 24%. The patient was anticoagulated (international normalized ratio (INR) 2-3).

**Table 1:** Laboratory results

FBC	Normal (including Hb & plts)
U&Es, LFTs	Normal
PT, APTT, Fib	Normal
Rheumatoid factor	Positive (24 IU/mL)
ANA	Positive
Lupus anticoagulants	Positive
Cardiolipin IgG/IgM	Negative
Antithrombin III	Negative
Protein C/Protein S	Normal
APC resistance	Normal
Prothrombin gene	Negative

Abbreviations: FBC, full blood count; U&Es, urea and electrolytes; LFTs, liver function tests; PT, prothrombin time; APTT, activated partial thromboplastin time; Fib, fibrinogen; ANA, antinuclear antibody; Ig, immunoglobulin; APC, activated protein C.

**Figure 3:**
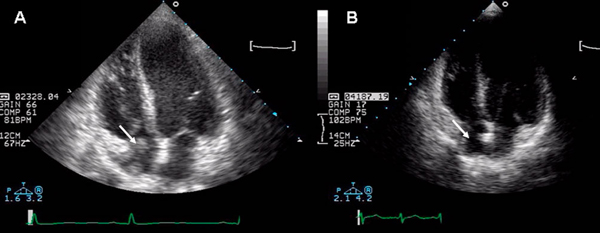
Four chamber view echocardiogram - pre (A) and post (B) treatment - showing resolution of the thrombus (arrow).

One year later, the patient was significantly better (oxygen saturation 95% in room air). Repeat echocardiography revealed resolution of the thrombus (Figure 3). Transcatheter closure of the PFO was performed but she remains anticoagulated due to the persistence of lupus anticoagulants and the TIVAD.

## Discussion

Our patient had worsening hypoxaemia and a paradoxical thromboembolic event in the context of a TIVAD, a PFO and lupus anticoagulants. Initially, the hypoxaemia was attributed to an infective exacerbation, but other diagnoses were later considered as oxygen saturation did not improve with her FEV_1_. These included interstitial lung disease (in view of the RA) and pulmonary embolism (a rare diagnosis in CF with few reported cases - all in the presence of a TIVAD [4,9]). A PFO with right-to-left shunting was later confirmed, demonstrating that this is possible without significant pulmonary hypertension. We speculate this was a result of tricuspid valve obstruction from the thrombus [10]. Additionally, manoeuvres such as Valsalva and coughing are known to increase the shunt size, which are likely to have been important in this patient [11]. We know of no other association in the literature of thrombus formation and the persistence of a PFO. Despite resolution of the thrombus with anticoagulation, transcatheter closure of the PFO was still performed because her persistent procoagulant state was considered to carry a significant risk of a further cerebrovascular event.

This was an unusual cause of hypoxaemia but should be considered when patients (especially with TIVADs) do not respond to conventional therapy. PFOs are not uncommon (incidence ~27% in the general population [12]) and lupus anticoagulants are present in 5% of the general population. Prior knowledge of her lupus anticoagulant status may have expedited the diagnosis but routine screening before TIVAD insertion is controversial as the presence of lupus anticoagulants do not predict a first thrombotic episode [13]. Furthermore, the efficacy of primary thromboprophylaxis for TIVADs is questionable; a recent consensus document advised against this in children as the available evidence does not show any benefit [14]. However, TIVADs are an independent risk factor for thrombosis (prevalence ~5% [15]) and therefore it is suggested in CF literature that thrombophilia screening should be considered [2].

## Conclusion

This case highlights the need to be vigilant to other causes of hypoxaemia in chronic respiratory diseases when patients are disproportionately hypoxaemic in relation to their lung function. TIVADs should always be considered as potential sources of thrombosis and a right-to-left shunt needs to be carefully excluded when investigating hypoxaemia not explained by the more common sequelae of the underlying condition.

## Abbreviations

CF: cystic fibrosis; CFTR: cystic fibrosis transmembrane conductance regulator; CT: computed tomography; FEV_1_: forced expiratory volume in 1s; INR: international normalized ratio; KCO: diffusing capacity of carbon monoxide corrected for loss of alveolar volume; PFO: patent foramen ovale; RA: rheumatoid arthritis; TIA: transient ischaemic attack; TIVAD: totally implantable venous access device.

## Consent

Written informed consent was obtained from the patient for publication of this case report and any accompanying images. A copy of the written consent is available for review by the Editor-in-Chief of this journal.

## Competing interests

The authors declare that they have no competing interests.

## Authors' contributions

NS collated the medical history and clinical investigations and also wrote the manuscript. HW, MH and KG were extensively involved in the management of the patient and made major contributors to writing the manuscript. RP provided expert haematological advice and contributed to the haematology aspects of the case report.
